# Personalised Prophylaxis in a Child with Haemophilia A and Type 1 Diabetes

**DOI:** 10.3390/clinpract11020041

**Published:** 2021-05-08

**Authors:** Maria Sol Cruz, Josefina Santillan, Julieta Lesser, Juan Pablo Ortiz, Laura Forzani

**Affiliations:** Hemophilia Foundation of Salta, Salta 4400, Argentina; josefinasantillan@hotmail.com (J.S.); jlesser@hotmail.com (J.L.); jpablogeo@gmail.com (J.P.O.); lauraforzani@hotmail.com (L.F.)

**Keywords:** haemophilia A, diabetes, simoctocog alfa, personalised prophylaxis, coagulation factor VIII

## Abstract

Poor management of either type 1 diabetes or haemophilia A can lead to complications such as organ dysfunction and haemarthropathy. Here, we describe the case of an 8-year-old boy diagnosed with severe haemophilia A shortly after birth. At 2 years old, he was also diagnosed with type 1 diabetes. After six years, the haemophilia treatment was changed from a plasma-derived factor VIII (FVIII) concentrate (octanate^®^, Octapharma, Lachen, Switzerland) to Nuwiq^®^ (simocotocog alfa, Octapharma, Lachen, Switzerland), a recombinant FVIII (rFVIII) product from a human cell line, which allowed for a personalised treatment schedule that supported good adherence. The dosing regimen could be reduced to two weekly rFVIII infusions. The patient has experienced no spontaneous bleeds since switching to rFVIII and shows no signs of joint damage after over seven years of FVIII prophylaxis. rFVIII was well tolerated, with no treatment-related adverse events observed. This case illustrates the importance of treatment personalisation for young patients and their families managing concomitant diseases.

## 1. Introduction

The current standard of care in haemophilia A (HA) is treatment with exogenous coagulation factor VIII (FVIII) concentrates, which may be plasma-derived (pdFVIII) or produced using recombinant cell technology (rFVIII). FVIII may be administered on-demand, to treat bleeds once they occur, or prophylactically at regular intervals in order to maintain a minimum level of FVIII in the plasma and prevent bleeds before they occur [1]. While the on-demand approach is effective at stopping bleeding, patients receiving such treatment often go on to develop long-term complications due to recurrent bleeding, with haemarthropathy being the most common [2]. The development of prophylactic regimens has greatly reduced bleeding rates and slowed the deterioration of joints; however, prophylaxis can have some drawbacks for patients with respect to time and potential venous access issues. Personalised prophylaxis takes into consideration patient-specific parameters in order to determine the optimal prophylaxis schedule for each patient [3]. This may lead to a reduction in the number of infusions and/or the total amount of FVIII administered. Personalised prophylaxis has also been shown to be effective in treating other rare blood disorders such as afibrinogenemia [4].

The optimisation of treatment is particularly important in patients with concomitant diseases requiring long-term and/or time-consuming treatment. Here, we describe a case of an 8-year-old boy with severe HA and type 1 diabetes mellitus, for whom HA treatment was successfully optimised in order to ease his treatment burden.

## 2. Case Report

**Figure 1:**
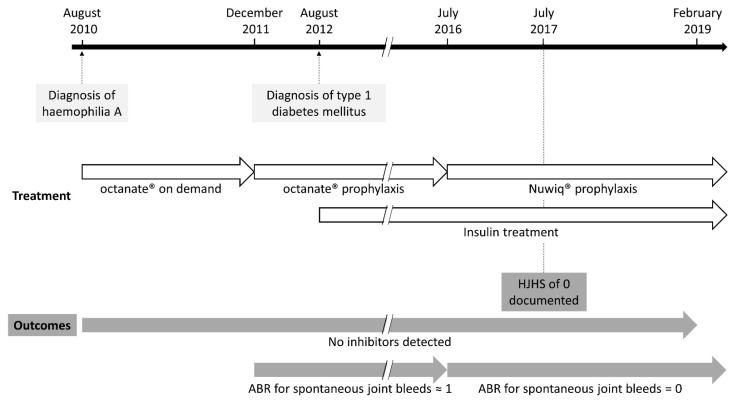
Timeline of key events and outcomes. Timeline of key diagnoses (light grey boxes), treatments (open arrows) and outcomes (dark grey arrows and boxes). ABR, annualised bleeding rate; HJHS, Haemophilia Joint Health Score.

The patient was born prematurely in June 2010 at 7 months of gestation. His older brother and one of his mother’s cousins were known to have HA; the mother is an HA carrier with an FVIII activity (FVIII:C) of 36%. He was referred to the Haemophilia Foundation of Salta, Argentina, in August 2010, at two months of age, following a diagnosis of HA (Figure 1).

The patient had experienced multiple bleeding episodes in the two months between birth and HA diagnosis. Based on family history and his clinical phenotype, HA was suspected and FVIII:C assays were performed. These showed FVIII:C was <1%, which confirmed the diagnosis of severe HA.

After referral to our centre, the boy and his family received education and training about HA and diabetes and how to deal with the impact of these chronic diseases on the lives, not only of the boy, but of the whole family. Psychological support was provided on an ongoing basis until the boy reached six years of age.

As HA is a disease caused by a gene defect, there is currently no cure and life-long treatment is required. Directly after diagnosis, the boy started on-demand treatment with pdFVIII concentrate stabilised with von Willebrand factor (octanate^®^). This concentrate has demonstrated excellent efficacy and low immunogenicity in previously untreated patients (PUPs). In December 2011, at 18 months of age, the prophylactic dose was increased to 500 international units (IU) (37.3 IU/kg body weight) three times weekly. Inhibitor development was monitored every 6 months; all tests were negative (<0.6 Bethesda units/mL).

In August 2012, he was additionally diagnosed with type 1 diabetes mellitus after experiencing symptoms including polyuria and fainting from hypoglycaemia. His parents confirmed the absence of a family history of diabetes. Insulin treatment was initiated immediately following the diagnosis.

Blood glucose was measured at least eight times a day as the levels depend on many factors (food intake, exercise, mood, etc.) and are challenging to predict. He tended to have nocturnal hypoglycaemia, and morning glucose levels were variable (65 to 200 mg/dL). Glycosylated haemoglobin (HbA1c) was measured once every 4–6 months; levels varied depending on the number of episodes of hyperglycaemia and typically ranged between 8.5 and 10%. His diabetes treatment schedule consisted of one subcutaneous injection of 16 U insulin glargine (Lantus 100^®^, Sanofi, Paris, France) in the morning and insulin aspart (NovoRapid FlexPen^®^, Novo Nordisk, Bagsværd, Denmark) on demand in order to keep blood glucose levels stable. Usually, 4–6 doses of on-demand insulin were required daily.

Given the complexity of the diabetes treatment, it was decided to modify the HA treatment. In July 2016, the patient was switched to prophylaxis with a 4th generation rFVIII produced in a human cell line (Nuwiq^®^), [5] in order to simplify therapy. In a study on personalised prophylaxis with rFVIII, over 50% of patients were able to receive prophylaxis at a frequency of twice weekly or less [6]. Prophylaxis with rFVIII also allowed a reduction to two weekly infusions in this patient, while offering additional advantages such as a shorter infusion time (1 mL per minute), a lower infusion volume (2.5 mL), and easier handling. rFVIII was obtained by the Haemophilia Foundation of Salta through a compassionate use programme until May 2017, when the product became officially available in Argentina.

Prophylaxis was started at 500 IU (approximately 24 IU/kg based on the patient’s body weight at that time) twice weekly, in accordance with his previous dosing of pdFVIII and clinical condition. In January 2017, the dose was adjusted to 1000 IU (approximately 43 IU/kg) twice weekly due to increases in both his levels of physical activity and body weight. The FVIII:C levels 30 minutes post-infusion of 1000 IU rFVIII were 38–39.5%. Whenever possible, infusions were given on the days when the most physical exercise was expected. In July 2018, the patient enrolled in the GENA-99 study, a prospective, multinational, non-interventional post-authorisation study to document the long-term immunogenicity, safety, and efficacy of rFVIII in patients with HA treated in routine clinical practice (NCT02962765).

Apart from being more convenient, rFVIII prophylaxis was highly effective at preventing bleeding in this patient. The patient had experienced approximately one joint bleed per year on prophylaxis prior to switching, with the right ankle being repeatedly affected. No spontaneous joint bleeds have been reported after the switch to rFVIII. Traumatic joint bleeds, which occurred occasionally when he played soccer, were successfully treated with two infusions of rFVIII and magnetotherapy. His Haemophilia Joint Health Score, a measure of the severity of persisting joint damage in patients with HA, was 0 in July 2017, i.e., there were no signs of permanent joint damage. He has experienced no bleeds, either spontaneous or traumatic, in the two years since switching to rFVIII. He has never reported episodes of epistaxis. Small haematoma occurred frequently following either minor trauma or insulin infusions but did not require any treatment. As a result, the patient did not feel limited in his daily activities by his haemophilia. He swims, plays soccer with his father, participates in physical education at school, and is a happy, cheerful boy who likes playing with friends.

He has shown good adherence to rFVIII and the treatment has been well-tolerated; no adverse events related to treatment have been observed. Importantly, no inhibitors against FVIII have been detected, either under pdFVIII treatment as a PUP or after switching to rFVIII. An inhibitor test (Nijmegen-modified Bethesda assay) was performed after 10, 20, and 50 exposure days and subsequently at 6-month intervals. All tests, including the most recent test in February 2019, showed a negative inhibitor titre (≤0.6 Bethesda units/mL).

## 3. Discussion

In this child suffering from both severe HA and diabetes, optimising HA treatment has greatly contributed to easing a busy treatment schedule involving many infusions, while maintaining effective bleed protection and allowing him to lead a normal, active life including regular sports activities.

This case demonstrates the feasibility of personalised prophylaxis for HA, even in patients with concomitant diseases who require intensive and time-consuming management. Prophylaxis is recommended for children with severe HA and has become the gold standard in most countries with adequate resources [7]. Several studies have demonstrated that prophylaxis with FVIII results in lower rates of both joint and overall bleeding, [8,9] better joint health, [8,9] and higher quality of life (QoL) [9,10] compared with FVIII administered on demand, and a recent systematic review has confirmed superior long-term outcomes [11]. However, prophylaxis requires a demanding treatment regimen [12] and ensuring adherence can be challenging.

Reported adherence rates of prophylaxis in HA patients vary widely and are generally suboptimal [13,14,15]. Poor adherence is clinically relevant because it results in unfavourable outcomes compared with good adherence: a number of studies have reported increased rates of overall and joint bleeds, [16] worse QoL, [16] and more chronic pain [17] in patients with poor adherence.

The good adherence to prophylaxis observed in the patient reported here may have been favoured by several factors. First, the switch to rFVIII therapy enabled a reduction in the number of infusions, as well as of the time required for infusion and handling, which is beneficial given the complex treatment schedule for the concomitant diabetes. Even in HA patients without comorbidities, non-adherence is often related to the time-consuming nature of prophylaxis with FVIII. The time that treatment takes has been repeatedly mentioned among the most common reasons for poor adherence in surveys, [12,18] and even among patients with excellent compliance, 30% named this as the most significant challenge [12]. This is confirmed by the finding that suboptimal adherence is typically characterised by changes in the timing of infusion (mostly from morning to evening) but not in dosing [15]. The reduced handling time is another improvement because the handling of FVIII is perceived to be inconvenient by patients, who feel that easier administration systems may facilitate better adherence [18]. Overall, the patient’s parents consider this rFVIII a “life-changer”.

In this context, it is important to note that good adherence has been found to be associated with treatment satisfaction [16]. The personalisation of treatment has the potential to address each individual patient’s needs and preferences, which is likely to increase treatment satisfaction. For patients treated with the rFVIII described in the case study, two options exist to personalise treatment, either based on individual pharmacokinetic (PK) parameters [6] or on a population PK model [19]. Together with the low infusion volume, excellent efficacy in the prevention of bleeding [6] and a demonstrated low immunogenicity, [20] this makes this rFVIII an attractive option to satisfy the diverse demands HA patients may have.

Second, the constant support provided by the Haemophilia Foundation of Salta is likely to have played a role. The patient’s parents have repeatedly stated that the Foundation has played an important role in helping them understand the disease and its management, and that the support they received has changed the way the patient sees haemophilia. It has been previously described that patient satisfaction with the healthcare team [16] and good relationships with haematologists/nurses [18] are associated with improved adherence. Importantly, the Foundation is also involved in the education of both the patient and his family on the necessity and the benefits of prophylaxis, another aspect that appears to be paramount for good adherence [12,13]. Lastly, the psychological support provided appears to have helped reduce negative emotions regarding the disease and fostered disease acceptance, which is known to be associated with better adherence [16,21]. This case therefore highlights how the availability of practical and psychological support can enable a patient and their family to be in full control of HA therapy.

## 4. Conclusions

In conclusion, this case illustrates the importance of collaboration between different medical specialties to provide personalised treatment and support for young patients and their families. The case demonstrates that it is feasible to administer FVIII prophylaxis using rFVIII or pdFVIII concentrates in patients with concomitant diseases associated with a high burden of care such as diabetes. Prophylaxis might also be particularly beneficial for older HA patients with diabetes because the number and severity of comorbidities, such as cardiovascular complications, tend to increase later in life. The optimisation of therapy enables patients with HA to lead a life with very few limitations and should therefore be encouraged in all patients.

## Data Availability

Data are contained within the article.
